# Retraction Note: Evaluating the predictive value of fetal doppler indices and neonatal outcome in late-onset preeclampsia with severe features: a cross-sectional study in a resource-limited setting

**DOI:** 10.1186/s12884-025-08163-1

**Published:** 2025-09-15

**Authors:** Eman Mohamed Ibraheim Moawad, Amr Samir Fouad Tammam, Maha Mohamed Mosaad, Hadeer Mashaal El Sayed, Adel Atef

**Affiliations:** 1 Department of Pediatrics, Faculty of Medicine, Cairo University, Giza, Egypt; 2 Department of Obstetrics and Gynecology, Faculty of Medicine, Cairo University, Giza, Egypt

**Retraction Note: BMC Pregnancy and Childbirth 22, 377 (2022)**

**https://doi.org/10.1186/s12884-022-04704-0**

The Editor has retracted this article. After publication, concerns were raised about the data and p-value calculation. The authors provided an explanation but did not provide their original data set on request. Post-publication review confirmed some of the concerns such as a very high number of severe pre-eclampsia cases, incorrect p-values and the indications for why caesarean sections were carried out in the control group, highlighting further concerns with the methodology. The Editor has therefore lost confidence in the integrity of this study. Eman Mohamed Ibraheim Moawad, Adel Atef and Amr Samir Fouad Tammam disagree to this retraction. Maha Mohamed Mosaad and Hadeer Mashaal El Sayed did not respond to correspondence regarding this retraction.

